# Correcting for intra-experiment variation in Illumina BeadChip data is necessary to generate robust gene-expression profiles

**DOI:** 10.1186/1471-2164-11-134

**Published:** 2010-02-24

**Authors:** Robert R Kitchen, Vicky S Sabine, Andrew H Sims, E Jane Macaskill, Lorna Renshaw, Jeremy S Thomas, Jano I van Hemert, J Michael Dixon, John MS Bartlett

**Affiliations:** 1 School of Physics, University of Edinburgh, 10 Crichton Street, Edinburgh, EH8 9AB, UK; 2 Endocrine Cancer Group, Edinburgh Cancer Research Centre, Institute of Genetics and Molecular Medicine, Crewe Road South, Edinburgh, EH4 2XR, UK; 3 Applied Bioinformatics of Cancer Group, Edinburgh Cancer Research Centre, Institute of Genetics and Molecular Medicine, Crewe Road South, Edinburgh, Edinburgh, EH4 2XR, UK; 4 Breast Cancer Research Group, Western General Hospital, Crewe Road South, Edinburgh, EH4 2XU, UK; 5 School of Informatics, University of Edinburgh, 10 Crichton Street, Edinburgh, EH8 9AB, UK

## Abstract

**Background:**

Microarray technology is a popular means of producing whole genome transcriptional profiles, however high cost and scarcity of mRNA has led many studies to be conducted based on the analysis of single samples. We exploit the design of the Illumina platform, specifically multiple arrays on each chip, to evaluate intra-experiment technical variation using repeated hybridisations of universal human reference RNA (UHRR) and duplicate hybridisations of primary breast tumour samples from a clinical study.

**Results:**

A clear batch-specific bias was detected in the measured expressions of both the UHRR and clinical samples. This bias was found to persist following standard microarray normalisation techniques. However, when mean-centering or empirical Bayes batch-correction methods (ComBat) were applied to the data, inter-batch variation in the UHRR and clinical samples were greatly reduced. Correlation between replicate UHRR samples improved by two orders of magnitude following batch-correction using ComBat (ranging from 0.9833-0.9991 to 0.9997-0.9999) and increased the consistency of the gene-lists from the duplicate clinical samples, from 11.6% in quantile normalised data to 66.4% in batch-corrected data. The use of UHRR as an inter-batch calibrator provided a small additional benefit when used in conjunction with ComBat, further increasing the agreement between the two gene-lists, up to 74.1%.

**Conclusion:**

In the interests of practicalities and cost, these results suggest that single samples can generate reliable data, but only after careful compensation for technical bias in the experiment. We recommend that investigators appreciate the propensity for such variation in the design stages of a microarray experiment and that the use of suitable correction methods become routine during the statistical analysis of the data.

## Background

DNA microarray technology has rapidly seduced scientists and clinicians with the ability to simultaneously measure the expression of tens of thousands of transcripts, enabling data-driven, holistic comparisons of groups or populations of cells, subtyping tissues, or predicting prognosis [1,2]. However, as with any method, sound experimental design is essential to generate robust results from microarray experiments, particularly given the issues of high dimensionality [3]. Sufficient care must be taken to identify and correct for sources of experimental bias alongside a cautious interpretation of the importance of reported differentially expressed genes [4].

Efforts to promote the routine formalisation and control of all stages of the experimental workflow have seen success and are increasingly promoted by journals and microarray data repositories [4]. More recent work suggests the need for the inclusion of more detailed information concerning the statistical treatment of data in order for results to be independently validated post-publication [5,6]. Such standardisation is essential to researchers wishing to re-analyse published data or combine multiple datasets in a meta-analysis. However the utility of these standards to the individual researcher gathering, analysing, and interpreting the data in the first instance is largely overlooked.

Despite all efforts towards standardisation, it is still not possible to account for all potential sources of variation in the experiment workflow; identical experiments performed at different sites have produced significantly different results [7-9]. Inconsistencies between results generated using different microarray platforms [8,10,11] or generations of array [12,13] have been highlighted and multiplicative, systematic biases have been shown to be introduced at many stages of the experimental process, even when using a single array platform [12].

The common practice of hybridising samples with no technical replication (i.e. one replicate of each sample per experiment) is a result of the relatively high cost of arrays, the perceived improvement in array manufacturing quality, and the difficulties of obtaining sufficient amounts of high quality mRNA from some clinical samples. This practice is fundamentally reliant on the assumption that the intra-experiment variability is of a small enough magnitude not to undermine the power of the assay to resolve interesting biological differences that may exist between predefined groups of samples. There is, however, mounting evidence [8,12,14-18] to suggest that this assumption may be flawed and that the technical variation between replicate samples should not be ignored.

A large amount of effort has been expended in assessing the reliability, reproducibility, and compatibility of results generated by a number of array platforms within and between laboratory sites. The microarray quality control (MAQC) project, a US Food and Drug Administration initiative [8], explored that the intra- and inter-platform consistency of microarrays using two reference RNA samples (a universal human reference RNA (UHRR) from Stratagene comprised of high-quality RNA from a mixture of 10 different human cell-lines (including breast) and a human brain reference RNA from Ambion) and primary samples processed on six microarray platforms at three different sites. The results of the MAQC and other studies highlight the fact that, despite the generally good consensus between results, data generated from different platforms, in different laboratories, by different investigators can be negatively affected by dataset-wide batch variation in the reported expression levels [8,10,19]. Several methods that can remove these batch differences have been proposed, tested, and evaluated. Batch effects have been shown to be minimised with correction methods such as, singular value decomposition [20], distance weighted discrimination [10], mean-centring [12], and ComBat [21].

It is slowly becoming accepted that batch effects are to be expected when combining data generated across different labs, by different researchers, or using different platforms [8,10-13]. There is a strong motivation to integrate multiple studies for meta-analyses that have increased statistical power afforded by larger sample-sizes, which can help to overcome basic limitations such as the inherent heterogeneity between biological subjects. Combined datasets can swell to include thousands of tumours and have been shown to lead to improved results and consensus findings [12,22-26].

Some researchers are now aware of bias arising due to analysis of samples at different sites or the use of different microarray platforms. The MAQC project [8], for example, was a multi-site and multi-platform comparison study, while others deal exclusively with the integration of data generated at geographically distributed locations. This study, to the best of our knowledge, is the first to assess the propensity for introduction of batch-processing effects at the same site and using the same protocol, making use of the multi-array Illumina BeadChip platform. We go further than the MAQC study by analysing both a commercial reference RNA and primary clinical material. This approach enabled us to demonstrate that it is possible to generate robust and reliable results, without the need for technical replication of starting RNA, but only when batch-processing effects are identified and suitably minimised. In this study we demonstrate compelling evidence for the existence of confounding batch-processing effects within a single experiment, using RNA prepared in the same laboratory, arrays hybridised and scanned at a single site, using a single protocol, and quantified on a single platform.

We investigated intra-experiment batch-processing variability on the Illumina BeadChip [27] platform, as multiple arrays on each chip allow an investigation of intra- and inter-run variation. This was achieved through the hybridisation of a sample of UHRR to a single array on each chip along with duplicate preparations of cRNA from fresh frozen breast tumour samples that formed part of a recent clinical study (Sabine VS, Sims AH, Macaskill EJ, Renshaw L, Thomas JS, Dixon JM, Bartlett JMS: Gene Expression Profiling of Response to mTOR Inhibitor Everolimus in Pre-operatively Treated Post-menopausal Women with Estrogen Receptor-Positive Breast Cancer, Submitted). Intra-experiment variation is common in other assays, such as quantitative RT-PCR (qPCR), where technical replicates and inter-plate calibrators are used to increase statistical resolution.

## Results

### Data quality

A qualitative measure of the performance of the BeadChips used in this study is provided by a measurement of the fraction of probes that are consistently called to be detected or undetected over all arrays. Analysis of detection consistency in the UHRR data in this study was comparable with the MAQC results [8] with 60-70% probes consistently called all-detected, and 80-90% genes consistently called as either all-detected or all-undetected, across all arrays in each run (data not shown). The coefficient of variation (CV) between and within the runs of the experiment was also consistent with the findings of the MAQC study, with a mean CV in quantile normalised data of around 7.5% (see Additional File 1). The Illumina arrays used throughout the MAQC study were the Human-6 (48K v1.0) BeadChips, which differ from the Human-8 (24K v2.0) BeadChips used in this study in terms of the number of features represented. The Human-6 (v1.0) chips contain twice the number of probesets available on Human-8 (v2.0), however a large percentage of these additional probesets have been found to be unreliable [28] and are all contained within a completely separate strip on the chip leading to normalisation issues [15]. The high level of agreement in the observed CV and detection calls suggest any differences between the array versions at the probe-level are small.

### Inter- and intra-run variation of the replicate UHRR samples

**Figure 1:**
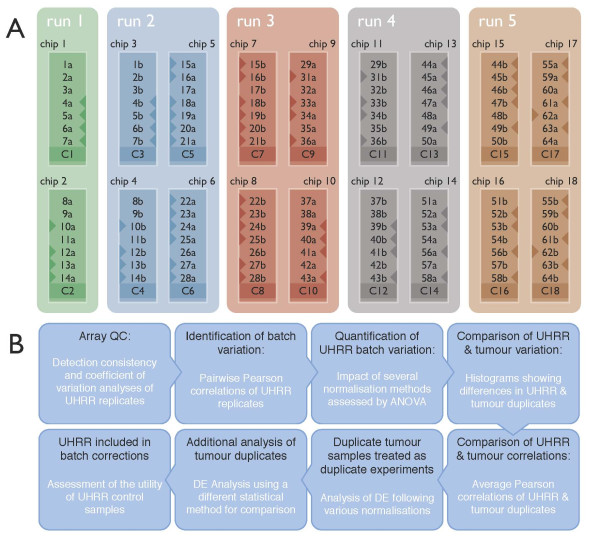
**Layout of samples on the Illumina BeadChips and flowchart of the analysis approach**. A, Illustration of the positions of samples on the 18 BeadChips, processed in five batches (also referred to as 'runs') corresponding to the five different days on which the samples were hybridised and scanned. UHRR samples are labelled as C1-18. Duplicate breast tumour clinical samples are labelled a and b. The pre- and post-treatment biopsy samples are identified by a triangle to the left and right of the sample IDs, respectively. B, Flowchart of analysis methods

**Figure 2:**
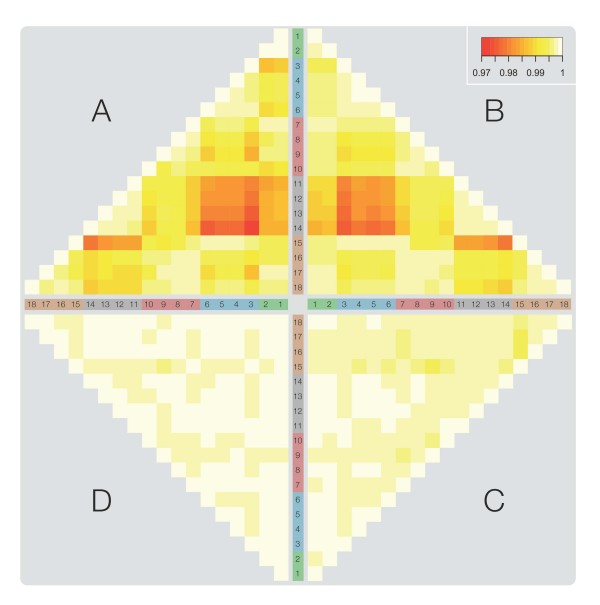
**Intra and inter-run variation in UHRR samples: Pearson-correlations**. Pairwise UHRR Pearson-correlation heatmaps highlight the batch differences, particularly between run 2 and run 4. Red cells correspond to ~97% correlation and white to 100% correlation. Batches and sample numbers are consistent with the colouring and labelling in Figure 1. All data were detection filtered, as described in methods. A = raw data; B = normalised; C = quantile normalised, plus mean-centring; D = quantile normalised, plus ComBat.

A clear batch-specific effect was observed in the raw data when the correlations of identical UHRR samples were assessed over all available pairs across the five runs processed on different days as illustrated in Figure 1. Generally, the level of correlation was high (>97%), however several clusters of samples were observed that corresponded to the batch in which the arrays were processed. In particular the samples in run 2 appeared to be very tightly correlated with each other but poorly correlated with samples in run 4 (Figure 2A). Quantile normalisation was found to have only a marginal improvement in the overall correlation of the samples and anomalies, such as that between runs 2 and 4, were conserved (Figure 2B). Only on application of specialised batch-correction methods, such as mean-centring (Figure 2C) and ComBat [21] (Figure 2D), were these run-specific disparities shown to be substantially reduced. The correlations (calculated using Pearson's rank-product) for quantile normalised data ranged from 0.9833-0.9991, whereas following a ComBat correction this was increased to 0.9997-0.9999.

The probe-wise standard deviations of the raw expressions were found to be consistently small across the UHRR arrays (mean = 0.28). Using the nested analysis of variance described in methods, 60% (mean value) of the variability was due to that between runs and less than 40% to that within each run. The magnitude of the variation was marginally increased by detection-filtering (mean = 0.31), which would be expected due to the preferential filtering of probes with low signal. The application of quantile normalisation had a positive effect, decreasing the standard deviation to half that of the raw data. However both after detection filtering and quantile normalisation the relative contributions of the inter- and intra-run components to the total standard deviation remained approximately unchanged. Of a selection of other normalisation methods, loess, and cubic-spline performed similarly to quantile and all of these methods out-performed simple median normalisation (Supplementary File 1). In all cases a further correction step is required after normalisation to correct for the batch effect.

**Figure 3:**
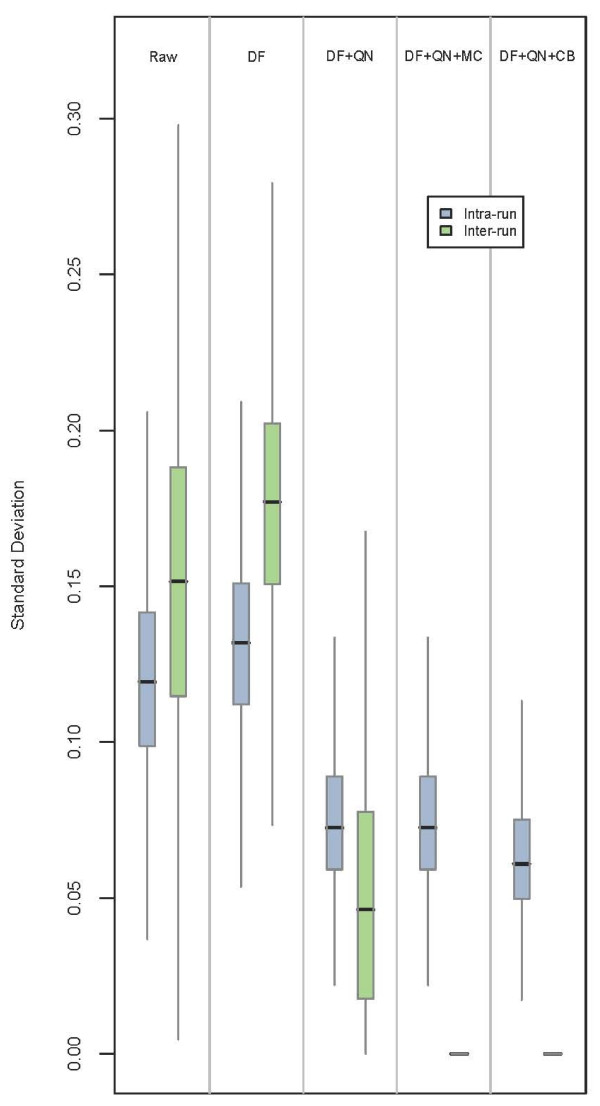
**Intra and inter-run variation in UHRR samples: Nested-ANOVA**. The results of a nested-ANOVA, quantifying the probe-wise components of variation corresponding to the within (blue) and between (green) batch variance. The model and calculation used are as described in methods. Effects on these standard deviations after detection-filtering (DF), quantile-normalisation (QN), mean-centring (MC), and ComBat (CB) are shown.

Both mean-centring and ComBat reduced inter-run variation to such an extent that it could no longer be accurately detected by the nested-Anova method (Figure 3). The only observable difference between the two methods was that the ComBat corrected data also showed a slight reduction in the intra-run component of variation (Figure 3). The sequence in which the data were quantile-normalised and batch-corrected appeared to produce only marginal differences in the resulting variance components; as a result, all remaining corrections using mean-centring and ComBat were performed after quantile normalisation for consistency and to comply with the statistical assumptions of the latter [16].

**Figure 4:**
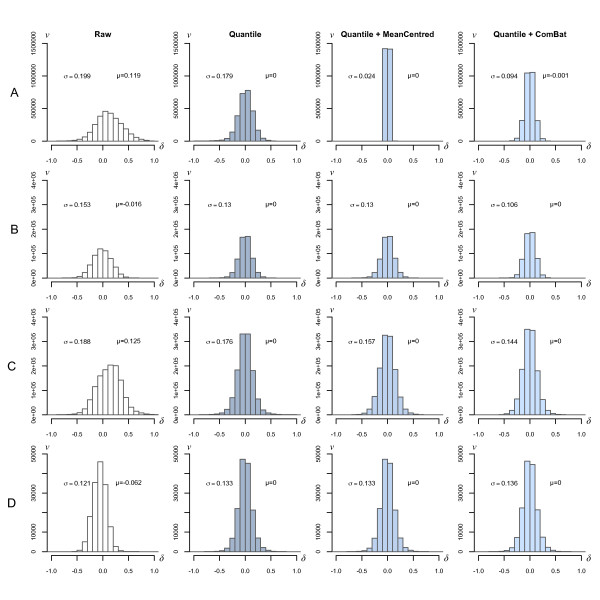
**Distribution of the differences between replicate pairs of intra- and inter run intensity measurements**. All possible combinations of differences between replicate pairs of UHRR controls and clinical samples were compared across the five runs. Axis labels represent the difference between duplicate samples (*δ*) on the x-axis, against frequency (*ν*) on the y-axis. Values on the left of each distribution represent the standard deviation and values on the right represent the mean of the measured differences. The four columns illustrate the effect of normalisation or batch correction on these differences. The four rows of plots illustrate both inter- and intra-run differences for both UHRR and tumour samples; row 'A' contains inter-run differences calculated between the 128 pairs of UHRR samples; row 'B' corresponds to intra-run differences between the 25 pairs of UHRR; row 'C' is the inter-run differences in the 56 pairs of tumour samples; and row 'D' contains data for the intra-run differences in 7 pairs of tumour samples in Run 5.

The differences in measured expression between all combinations of pairs of UHRR samples that straddled the five runs (128 pairs) were calculated for raw, quantile-normalised, mean-centred, and ComBat corrected data (Figure 4A). The distribution of differences in the raw data did not resemble the expected form of a gaussian centred at the origin; instead it was skewed towards the positive (mean = 0.199). This was largely corrected after quantile normalisation and subsequent application of mean-centring and ComBat further narrowed the distribution reflecting the previously observed improvement in correlation. Similar improvements were observed in the differences between samples that were processed in the same run (25 pairs, Figure 4B). A full illustration of the intra-run pairwise differences can be found in Additional File 2.

### Duplicate clinical breast-tumour samples

The sixty-three duplicate clinical samples provided a means to assess inter- and intra-run variation using samples more representative of those commonly analysed using microarray technology. The differences in the measured expressions between each of the duplicate pairs of the clinical samples that straddled the five runs (56 pairs) were calculated for raw, quantile-normalised, mean-centred, and ComBat corrected data (Figure 4C). As with the UHRR samples, moderate differences were observed between the raw expressions of duplicate hybridisations and quantile normalisation was found to reduce, but not eliminate, the differences between the duplicate samples. The distributions are similar to UHRR samples, although the raw data showed a clear positive skew that was again successively improved following quantile-normalisation, mean-centring, and ComBat, respectively. For completeness, intra-run distribution of differences between the duplicate samples was assessed for the seven pairs of samples in run five (Figure 4D).

**Figure 5:**
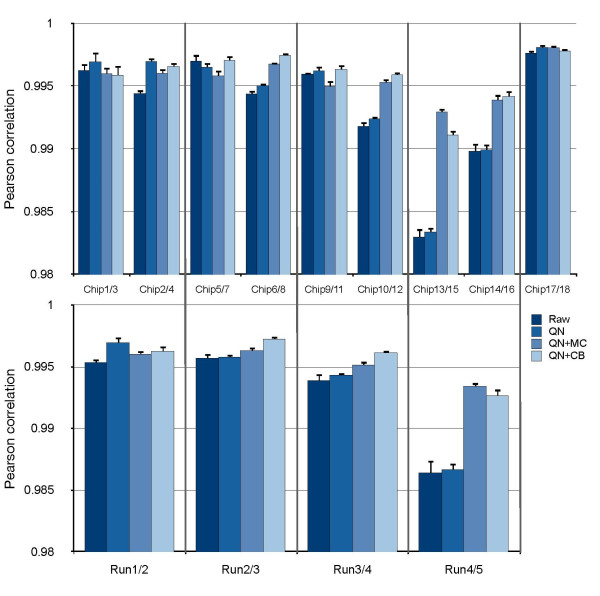
**Intra and Inter-run comparisons of clinical duplicates**. Mean Pearson-correlations between replicate pairs of tumour samples (A and B) on different chips and runs. Colours denote the four different data types; raw, quantile normalised (QN), quantile normalised then mean centred (QN+MC), and quantile normalised then ComBat corrected (QN+CB). Expressions were generally highly correlated except in the chips straddling runs 4 and 5. ComBat is able to correct for a significant amount of this difference. Error bars represent the standard error.

Pearson rank-products were calculated to assess the correlation between the duplicate samples. As with the UHRR the clinical samples were generally very highly correlated (>98%), although the samples on BeadChips 13/15 and 14/16 were found to be less similar than the others (Figure 5); this is consistent with the effect observed in run 4 using the UHRR. Batch correction by either mean-centring or ComBat increased the correlation for all samples except for two arrays on BeadChips 1/3 in the first run and all arrays on BeadChips 17/18 in the final run.

### Comparing duplicate tumour samples as a repeated dataset to assess reproducibility of gene-lists

Of the 63 duplicate, paired clinical samples obtained from matched-biopsies before and after treatment with the mTOR inhibitor RAD001, 42 were of sufficient quality to be used in an analysis to reveal differentially expressed genes (Sabine VS, Sims AH, Macaskill EJ, Renshaw L, Thomas JS, Dixon JM, Bartlett JMS: Gene Expression Profiling of Response to mTOR Inhibitor Everolimus in Pre-operatively Treated Post-menopausal Women with Estrogen Receptor-Positive Breast Cancer, Submitted). Using these samples we further assessed the impact of the intra-experiment variation in terms of the differences between lists of differentially expressed genes reported by each half of the duplicate samples. The hybridisation plan for the 21 pairs of pre- and post-treatment samples in each duplicate-group is illustrated in Figure 1; in the figure, triangles to the left of the sample represent pre-treatment samples and triangles to the right represent post-treatment samples. The first hybridisation of each duplicate sample is represented by a trailing 'a' and the second represented by a trailing 'b'.

The 'A' and 'B' duplicate sample groups, containing the 'a' and 'b' hybridisations of each sample, respectively, were considered as two completely independent datasets (as they were processed on completely separate BeadChips) in order to assess the extent to which run-specific processing bias can influence the identification of differentially expressed genes. These datasets were independently filtered by detection calls, quantile-normalised, and, where stated, batch corrected by mean-centering or ComBat before generating lists of differentially expressed genes. Two BioConductor packages, *limma *and *siggenes*, were used to perform the statistical analyses (see methods).

**Figure 6:**
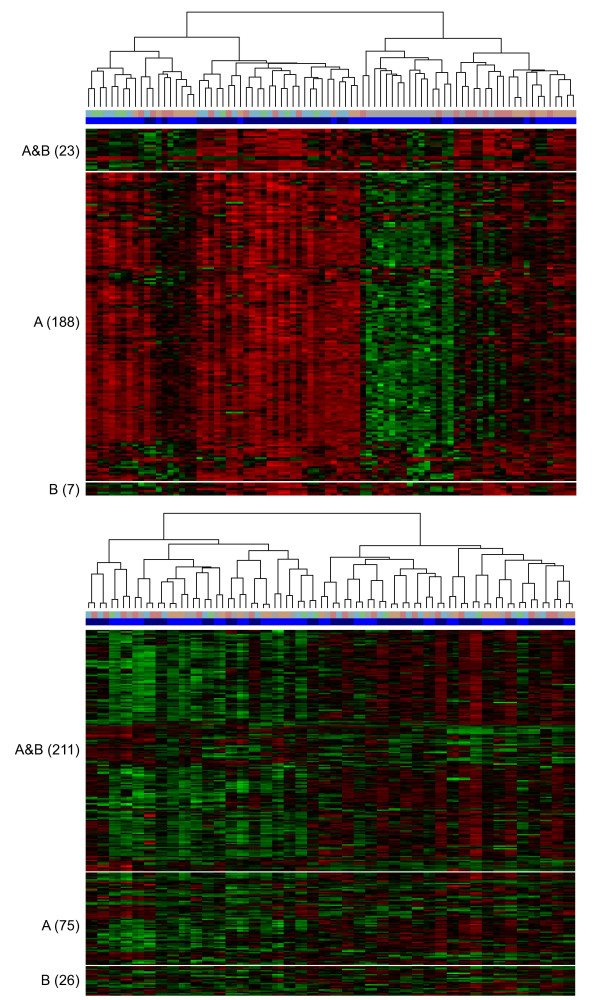
**Differentially expressed genes with duplicates treated as separate datasets**. Heatmaps of genes found to be differentially expressed in each of the A and B replicate datasets of samples and the overlap after quantile normalisation (top) and ComBat batch-correction (bottom). The batch in which each sample was present is denoted by bar beneath the dendrogram, in which the run-colours are consistent with those in Figure 1, and the sample-type is illustrated by the blue bar (light = post-treatment, dark = pre-treatment). The numbers of probes differentially expressed in both A and B ('A&B') or 'A' only and 'B' only are shown in brackets. Sample clustering (by complete linkage) in each heatmap was determined by only those probes in the 'A&B' group.

Using the same stringency in the assessment of differential expression (fold-change ±1.5, adjusted p-value of 0.01) and using quantile normalised data, many more probes were found to be differentially expressed between pre- and post-treatment samples in sample group A (192) than in group B (30). Following batch correction with ComBat the number of differentially expressed genes identified in the two groups was more consistent (260 and 211) and the overlap, in terms of probes reported in both groups, increased from just 11.6% to 66.4%, however the use of mean-centred data only increased the overlap marginally to 15.2% (Table 1, Figure 6, and Additional Files 3 and 4).

**Table 1:** Summary of comparing the duplicate tumour samples as a repeated dataset (A and B) to assess the reproducibility of gene-lists.

		A	B	A & B overlap	consensus (%)
**Limma**	**QN**	192	30	23	11.6
	**MC**	225	222	59	15.2
	**CB**	260	211	188	66.4

**SAM**	**QN**	214	40	30	13.4
	**MC**	240	238	65	15.7
	**CB**	265	218	193	66.6

**Limma + UHRR**	**QN**	205	31	24	11.3
	**MC**	8	92	7	7.5
	**CB**	144	119	112	74.2

**SAM + UHRR**	**QN**	224	42	32	13.7
	**MC**	17	100	12	11.4
	**CB**	149	125	117	74.5

Differentially expressed genes were identified using Limma and SAM as described in the text with quantile-normalisation (QN), mean-centring (MC), and ComBat (CB). The UHRR was used as an inter-batch calibrator.

Similar results were seen with less stringent criteria (fold-change ±1.2), which consequently led to larger numbers of probes, but similar proportions of overlapping probes were reported (data not shown). The analysis was repeated using significance analysis of microarrays (SAM) at a predicted false discovery rate of 5% and generated very similar results to those obtained using *limma*, increasing the overlap between groups of samples from just 13.4% in quantile-normalised data to 66.6% following ComBat batch-correction. See Table 1 for a full summary of these results. The heatmaps in Figure 6 (and Additional File 4) also highlight how all the pairs of duplicate samples cluster together following ComBat correction and the clustering is far less affected by processing runs. The dependency between the choice of ComBat, mean-centring, or quantile normalisation on the number of genes identified as differentially expressed in each replicate group was very strong in both the *limma *and *SAM *analyses (*χ*^2^(2) p-value << 0.001).

In addition to these independent analyses, the A and B groups were combined to create a third group of samples, 'C'. This group was analysed for differential expression in the same way and the results summarised in terms of the number of genes reported in any one, or any combination, of the three lists. The percentage of genes consistently reported by *limma *as differentially expressed in all three groups after ComBat correction was 41.3% compared to 11.2% after quantile normalisation alone and 12.0% after mean-centering (Additional File 3). The percentage of genes identified in the pooled group C compared with those consistently reported in all three groups increased from 44.2% after quantile normalisation to 90.1% after ComBat. Again, very similar results were observed using SAM (Additional File 3).

These analyses were repeated using the UHRR as inter-batch calibrator, designating it as a covariate in both the mean-centring and ComBat corrections. The inclusion of UHRR during quantile normalisation produced only a small difference in the number of differentially expressed genes identified in each of the three sample groups. However, the inclusion of the UHRR as a covariate in the mean-centring and ComBat corrections gave very different results. In both methods there was a large reduction in the total number of genes reported in each list, in terms of the consensus between the A and B groups, the agreement dropped to 7.5% following mean-centring, but increased to 74.1% after correction by ComBat (Table 1 and Additional File 3). The dependency between choice of batch correction method and number of genes reported in either replicate group was stronger when UHRR was included in the correction in both the *limma *and *SAM *analyses (*χ*^2^(2) p-value <<< 0.001).

## Discussion

Batch-processing effects in microarray experiments are commonly encountered when combining datasets from different studies, different labs, or different technologies. In this study we have demonstrated that batch effects can arise within a single study, at a single lab, using a single technology and that these can have a significant impact on reported gene-lists.

The magnitude of the variation in the observed expression of replicate samples derived from the UHRR in this study is consistent with that reported in other studies assessing the quality of microarray data, such as the MAQC [8]. We have also shown that the correlation of replicate UHRR samples is similar to that between duplicate pairs of samples derived from clinical breast-tissue biopsies and that this correlation is generally high. However, when duplicate groups of clinical samples were independently analysed to identify differentially expressed genes the consistency of the resulting gene-lists was found to be very poor. The predicted false discovery rate of 5% using SAM was far lower than the observed proportion of genes that failed to be consistently reported over the replicate analyses (~87% after quantile normalisation, ~30% after ComBat correction). Whilst these two values are not directly equivalent, our results suggest that the predicted FDR may imply greater consistency than would be measured if duplicate samples are available. Specialised corrections for run-bias were more successful in reducing the magnitude of variability attributed to the inter-run batch effect in both UHRR and clinical samples. The reliability of results generated from the duplicate clinical samples was also greatly increased following batch-correction with a much greater proportion of genes consistently reported as differentially expressed in both sets of samples.

### Use of single samples

There are many stages of sample-processing prior to conducting any gene-expression experiment and each is vulnerable to the introduction of systematic processing errors [12]. Opportunities to quantify this variation, prior to the microarray data analysis itself, are extremely limited and generally the only available option is an assessment of RNA quality. Other methods of quantifying gene expression, that are equally susceptible to the introduction of processing error, rely on the use of technical replicates to minimise confounding variation and maximise statistical resolution to the biological processes under investigation [29]. In this respect the routine practice of analysing each expression array sample as a singleton, regardless of the amount of RNA loaded, is an unusual scientific approach. Whilst BeadChip technology has a degree of built-in replication (approximately 30 randomly positioned beads, to which are attached ~700,000 identical copies of a gene-specific probe [27]), this is no substitute for biological replicates, especially when a large degree of the observed error can be attributed to noise at the sample level, rather than at the probe level.

In the context of primary breast tumour samples, which have been repeatedly shown to have highly heterogeneous mRNA expression profiles, there is much greater variation between the RNA profiles from different individuals than within tumours [30]; either when comparing different tumour sections, biopsies and the tumour or FFPE and frozen [31], which effectively characterises the 'intrinsic profile' of subtype classification. On this basis and the grounds of cost and scarcity of primary material it could be argued that replicates are unnecessary. However a lack of replicates limits the investigator in terms of their ability to assess whether the observed variation is of biological or technical origin and the extent to which it influences the resulting gene-lists. In this respect both biological and technical replicates are desirable to allow generated data to be screened for bias and batch-correction applied where appropriate. This is particularly important in the clinical setting if samples for large trials are processed in multiple labs.

Using the duplicate-experiment approach we were able to demonstrate that single samples can generate reliable data, particularly when batch correction is performed to minimise processing bias. However the genes reported to be differentially expressed in the pooled duplicate samples in group C were more robust in terms of their agreement with those identified in groups A and B, especially following batch-correction.

### Use of UHRR controls

In addition to the technical replicates commonly used in other assays, in cases where the execution of the assay is split into several runs, it is very common for an inter-run calibrator to be used to quantify the variation introduced by the splitting of the experiment and to normalise for it. Despite the UHRR samples used in this study showing very similar variation and correlation to that previously reported, we found that the samples were of limited utility as predictors of the batch variation amongst the clinical samples. However the replicate UHRR samples were found to slightly improve the consensus between the results of the duplicate experiments when used in conjunction with the ComBat correction.

Although the UHRR has been reported to be useful as a standard for microarray experiments and suitable for monitoring the performance of genome-wide expression platforms [8,9,32], it has also been reported to not be a suitable representative as a normal sample for colon epithelial RNA [32]; similarly, the UHRR does not contain breast tumour RNA (only that from a breast cancer cell line among a pool). A more reliable control sample with which to improve the batch-correction might be provided by an mRNA sample more representative of that under investigation; in this case, a pool of tumour RNA rather than the UHRR. We found that the pre-treatment samples were good predictors of the batch variation amongst the post-treatment samples and so would likely make a better control (for normalisation) than the UHRR.

### Experimental design

There is no reason to believe that the batch-processing effects observed here are limited to the Illumina BeadChip platform. Many previous investigations of other platforms have postulated potential factors responsible for the introduction of processing errors in microarray experiments [12,33-35]. Other experiments at our facility using the more recent Illumina Human HT-12 and Mouse Ref 8 BeadChips exhibit similar batch effects to those in this study; samples are observed to cluster preferentially with others processed in the same run, rather than by the biological differences between them, even after quantile normalisation (data not shown). Specialised batch-corrections appear to remove the bias, however without replicates such as those described in the current study, this cannot be fully evaluated.

Regardless of the platform chosen, it is clear that compensation for processing variation is beneficial and can only be achieved by incorporating the design of the experiment into the downstream data analyses. If all pre-treatment samples had been processed in one batch and all post-treatment samples in a second batch, it would not be possible to rule out confounding differences between treatment and batch processing. 'Real' differences due to the common variable of interest may have been partially or completely obscured by the batch effect. Design oversights of this type are beginning to be highlighted [36] and demonstrate the need to record the batches or processing runs in which data is generated. Some raw files contain metadata, such as the date in which they were generated, embedded within them. Acknowledgement and identification of the propensity for processing variation can be used to maximise the efficacy of batch-correction methods through a more informed design of the hybridisation-plan that includes, for example, randomisation and/or blocking of samples.

Our results support the notion that analysis of gene expression data should begin with an evaluation of batch effects. If the possibility of batch effects has been anticipated and confounding factors separated, then it should be possible to remove the bias to generate more robust results. As with other studies, ours is limited by the samples that were used in the evaluation of processing variation. We would have liked to test the applicability of our findings in other published datasets, however we were unable to find comparable datasets that include technical replicates and details of hybridisation 'batches' in the existing data repositories. In terms of cost and practicalities it is understandable why most researchers do not perform replicates in clinical studies, our results indeed suggest they may not be necessary; however providing a hybridisation plan along with the raw data, would make the processing of data more transparent.

## Conclusions

In summary, intra-experiment bias can distort the findings of gene expression studies. Replicate samples were found to be beneficial in both the identification and reduction of processing bias and lead to increased consensus in reported gene-lists, especially following specialised batch-corrections. We conclude that single samples can generate reliable data, although an appreciation for sources of intra-experiment variation during the design of the experiment is required to maximise the efficacy of specialised corrections in order to minimise susceptibility to potentially confounding intra-experiment batch-effects. Finally, based on the discrepancy between the lists of differentially expressed genes in each group of duplicate tumour samples, the observed rate of falsely-reported genes was consistently and significantly larger than that predicted by SAM. Therefore, based on the results of this study, a healthy degree of skepticism is advised when interpreting published results of microarray experiments that do not include validation by technical replication or, preferably, by another technique such as qPCR. In the absence of large numbers of biological replicates, it is our opinion that technical replication should be encouraged in order to provide robust, reliable, and credible expression-profiles.

## Methods

### Samples

In order to compare the consistency of gene expression profiles between and within processing runs a single sample of Universal Human Reference RNA (UHRR; Stratagene, Stockport, United Kingdom) was added to eighteen Illumina HumanRef-8 v2 Expression BeadChips. The remaining seven arrays on each chip were used to analyse the response to an mTOR inhibitor, Everolimus, in pre-operatively treated post-menopausal women with oestrogen receptor-positive breast cancer. From each extraction 100 ng RNA was amplified and biotinylated using Illumina^® ^TotalPrep RNA Amplification Kit (Ambion) and quantified on a Bioanalyser 2100. 750 ng cRNA per sample was hybridized to Illumina HumanRef-8 v2 Expression BeadChips (Illumina, Cambridge, United Kingdom) using Whole-Genome Expression Direct Hybridisation kit (Illumina) and scanned with a BeadStation 500GX (Illumina). Full details of the sample biopsies taken at diagnosis and at surgery were as previously described (Sabine VS, Sims AH, Macaskill EJ, Renshaw L, Thomas JS, Dixon JM, Bartlett JMS: Gene Expression Profiling of Response to mTOR Inhibitor Everolimus in Pre-operatively Treated Post-menopausal Women with Estrogen Receptor-Positive Breast Cancer, Submitted). The duplication of the clinical samples was performed after labelling and labelled samples were stored as per the manufacturer's recommendations.

All raw gene expression files, clinical annotation and R scripts used to perform the analysis are publicly available from the caBIG supported Edinburgh Clinical Research Facility Data Repository https://catissuesuite.ecmc.ed.ac.uk/caarray/.

### Statistical Methods

A summary work flow of the analysis approach is given in figure 1. Gene expression changes were compared before and after RAD001 treatment and between responders and non-responders using Bioconductor [37] algorithms implemented in the statistical programming language, R [38]. Illumina probe profile expression data were normalised using quantile normalisation and corrected for batch processing effects using mean-centring [12] and ComBat [21]. Unless otherwise stated, the UHRR and breast tumour samples were normalised separately and the UHRR samples were not included as a covariate in the mean-centring or ComBat corrections. Genes differentially expressed between pre- and post-treatment samples were identified using *limma *[39] and SAM [40]. For the analysis using the *limma *package, genes were defined as being differentially expressed after satisfying a minimum fold-change of ±1.5 and a maximum, Benjamini-Hochberg adjusted, p-value of 0.01. For the SAM analysis (using the *siggenes *package), the differentially expressed genes were selected at a maximum predicted false discovery rate of 5% and the same minimum fold-change of ±1.5. Paired statistical tests were performed in both the *limma *and the SAM analyses. Hierarchical clustering of samples and probes for the creation of all heatmaps was performed using complete linkage and similarities calculated according to the method described in [41].

Data were filtered, where specified, using the detection confidence reported by Illumina's BeadStudio software- determined for each bead based on the expressions of internal control probes, local background intensity, and the uniformity of the reported intensity of the bead. The filtering was performed prior to normalisation such that probes with a detection confidence less than or equal to 80% in more than 25% of the samples were removed from further analysis.

We applied a linear additive model to UHRR expression data on the log-scale to estimate the inter- and intra-batch variance contributions. These contributions are assumed to be independent and randomly drawn from log-normal distributions. As all factors meet in unique combinations a nested, or hierarchical, variance model is individually applied for each gene such that the model of the measured expression, *X*_*ij*_, of each probe is defined as

where *μ *is the geometric-mean expression of the gene from the UHRR population, *A*_*i *_is the random effect attributed to the *i*^th ^batch, and *ε*_*ij *_is the random measurement error attributed to the j^th ^array in the i^th ^batch. Finally, *b *is the total number of batches and *n *the number of replicate samples in the corresponding batch. The variance of any given observation, *X*_*ij*_, is σ^2^_*A *_+ σ^2^; these components represent the inter-batch and intra-batch variance respectively. The estimation of σ^2^_*A *_and σ^2 ^is performed independently for each gene as stated in [42].

## Authors' contributions

VSS conceived the study and processed the samples. RRK and AHS analysed the data. AHS, JIvH, and JMSB supervised the work. EJM, LR, and JMD performed biopsies and collected the clinical material. JST quality-assured the diagnostic material. RRK and AHS drafted the manuscript. All authors read and approved the final manuscript.

## Supplementary Material

Additional file 1 **Coefficient of variation amongst replicate UHRR samples. **Two box and whiskers plots of the coefficient of variation (CV) of the replicate UHRR samples. The first plot (A) shows the experiment-wide CV of the UHRR samples. The left-most of the four main sections shows the CV of the raw (detection filtered) data, to the right of this is the CV after four popular normalisation algorithms; quantile, loess, cubic-spline (qspline), and median. The final two segments show the CV after batch-correcting each normalised dataset using either mean-centring or ComBat. In the second plot (B), from the left, the first four segments contain five box-plots illustrating the CV within each of the five runs; the four segments containing raw (white), quantile-normalised (dark-blue), mean-centred (lighter-blue), and ComBat-corrected (pale-blue) data respectively. All data were detection-filtered prior to analysis. The right-most segment shows the experiment-wide CV of the UHRR (coloured as the previous segments) calculated with no consideration of the individual runs.
Click here for file

Additional file 2 **UHRR inter-run pairwise differences**. Pairwise differences between each of the five runs calculated using UHRR samples for raw, quantile-normalised, mean-centred, and ComBat-corrected data. Click here for file

Additional file 3 **Number of differentially expressed genes identified in replicate analyses**. Numbers of genes reported to be differentially expressed after standard analysis (quantile normalisation) (left), after a standard analysis with mean-centring (middle), and after a standard analysis augmented with the ComBat batch correction (right). A and B refer to the results from independent analyses of the duplicate sample groups while C refers to the results from the pooled duplicate samples. The rows of Venn diagrams illustrate the results with (i) limma, (ii) SAM, (iii) limma using UHRR, and (iv) SAM using UHRR. Click here for file

Additional file 4 **Heatmaps**. Full heatmaps of quantile normalised, quantile normalised plus mean-centred and quantile normalised plus ComBat data, including probe and sample annotations. Click here for file
