# Supplementary figures and images for: Correcting for intra-experiment variation in Illumina BeadChip data is necessary to generate robust gene-expression profiles
Source: BMC Genomics. 2010 Feb 24;11:134. doi: 10.1186/1471-2164-11-134 (PMC2843619; doi:10.1186/1471-2164-11-134)

CV (%)

0 5 10 15 20 25

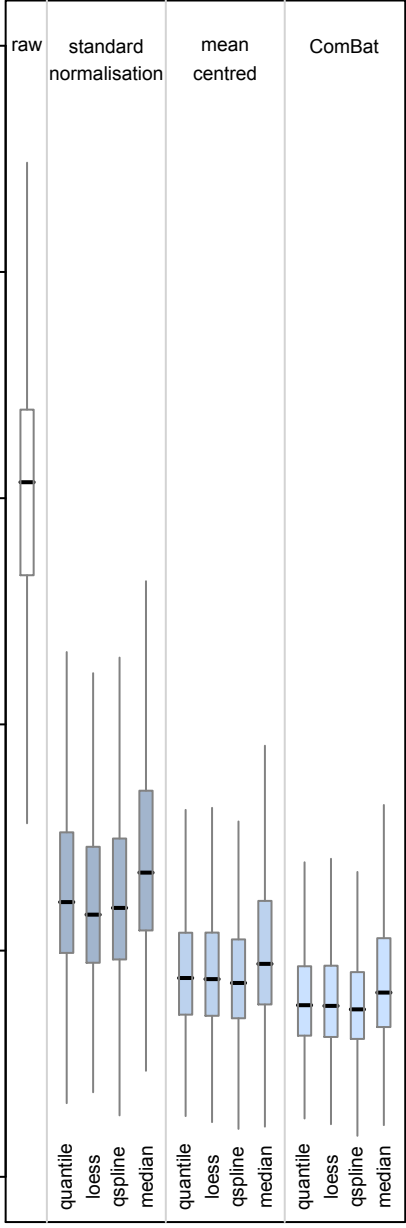

# CV

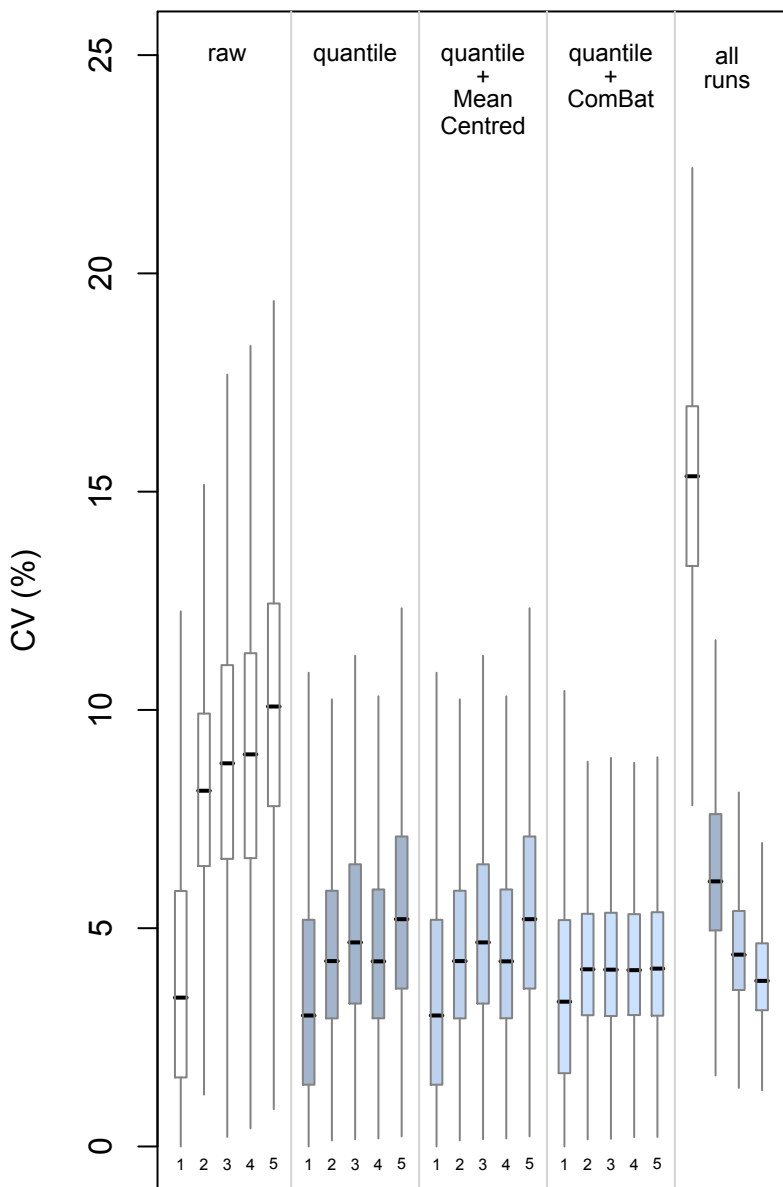

Supplement: Additional file 1 — Coefficient of variation amongst replicate UHRR samples. Two box and whiskers plots of the coefficient of variation (CV) of the replicate UHRR samples. The first plot (A) shows the experiment-wide CV of the UHRR samples. The left-most of the four main sections shows the CV of the raw (detection filtered) data, to the right of this is the CV after four popular normalisation algorithms; quantile, loess, cubic-spline (qspline), and median. The final two segments show the CV after batch-correcting each normalised dataset using either mean-centring or ComBat. In the second plot (B), from the left, the first four segments contain five box-plots illustrating the CV within each of the five runs; the four segments containing raw (white), quantile-normalised (dark-blue), mean-centred (lighter-blue), and ComBat-corrected (pale-blue) data respectively. All data were detection-filtered prior to analysis. The right-most segment shows the experiment-wide CV of the UHRR (coloured as the previous segments) calculated with no consideration of the individual runs. [file 1471-2164-11-134-S1.PDF]

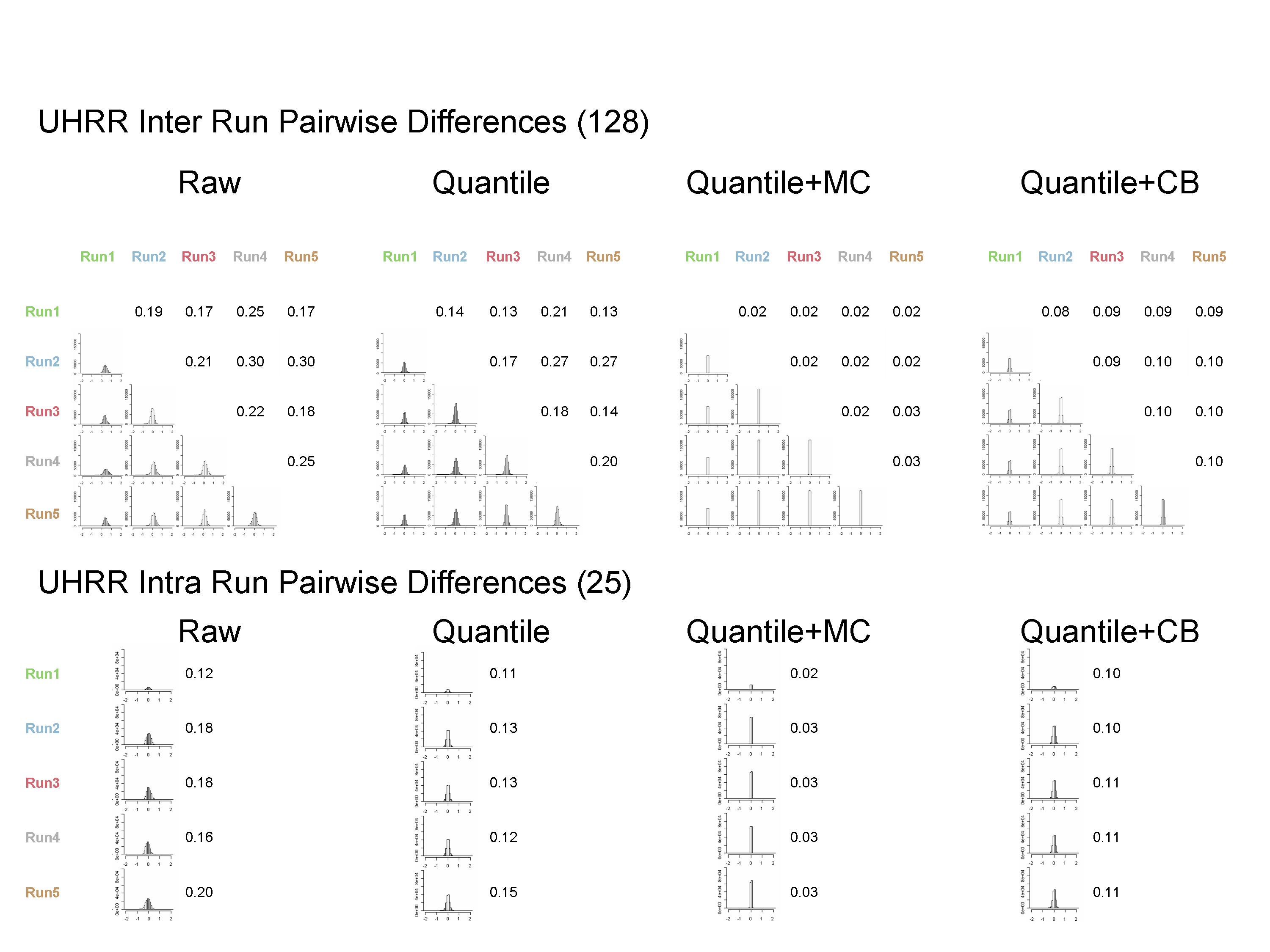

Supplement: Additional file 2 — UHRR inter-run pairwise differences. Pairwise differences between each of the five runs calculated using UHRR samples for raw, quantile-normalised, mean-centred, and ComBat-corrected data. [file 1471-2164-11-134-S2.JPEG]

(i)

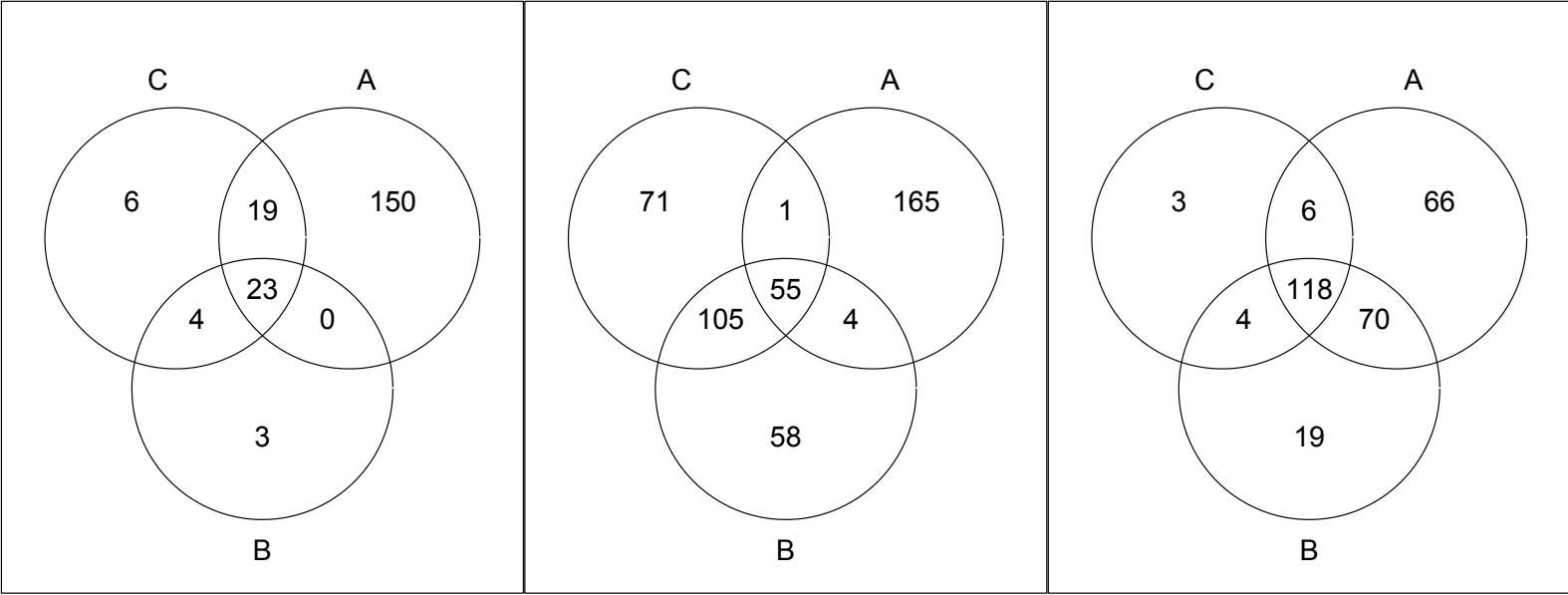

(ii)

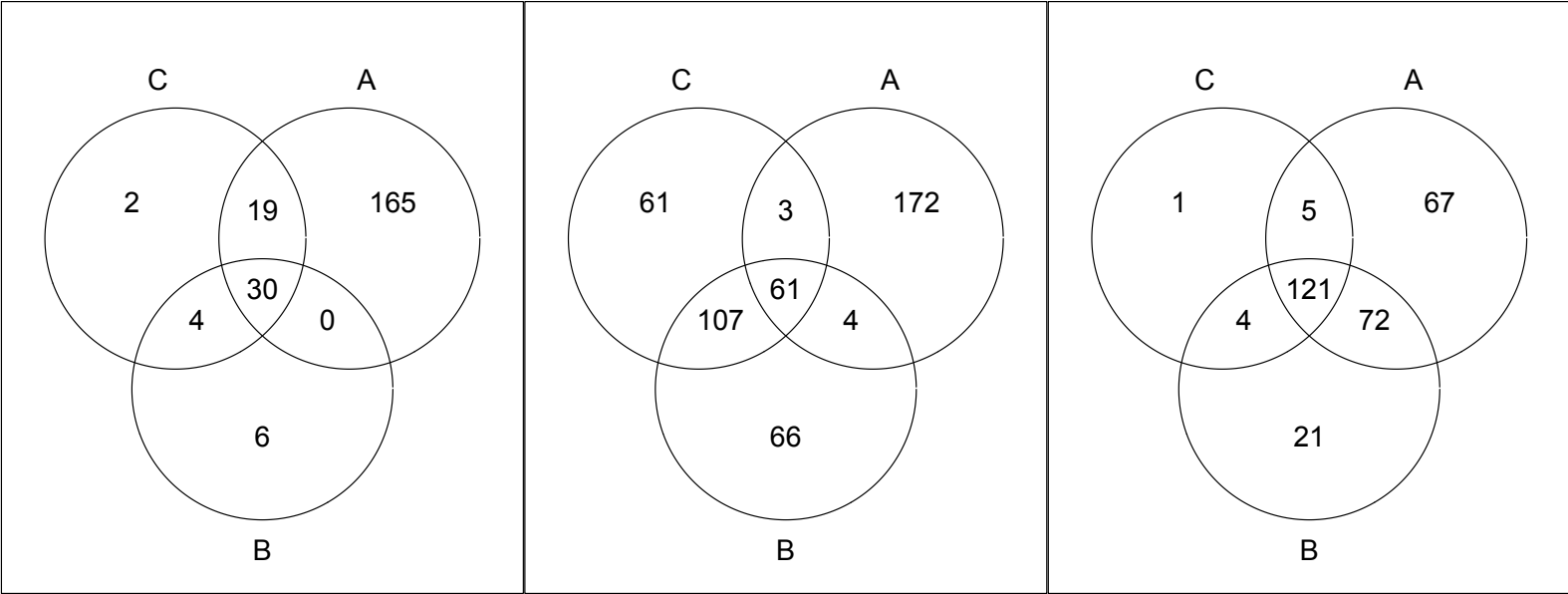

(iii)

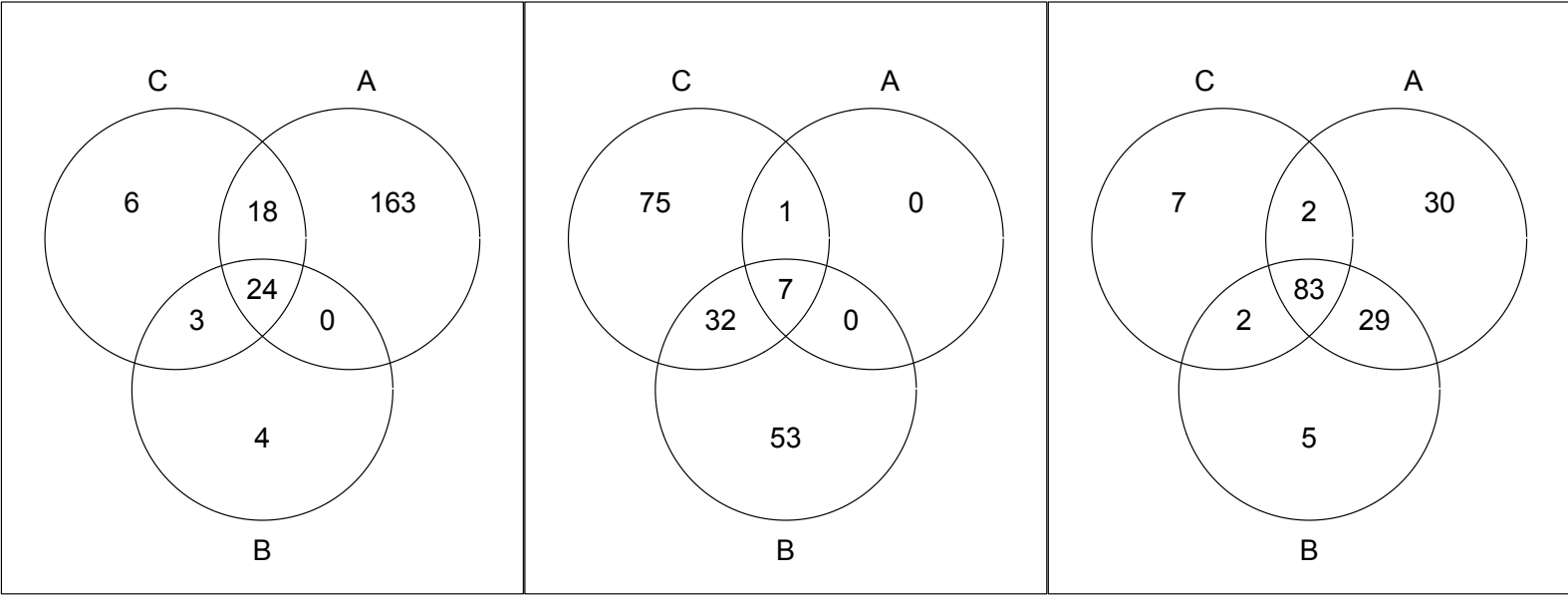

(iv)

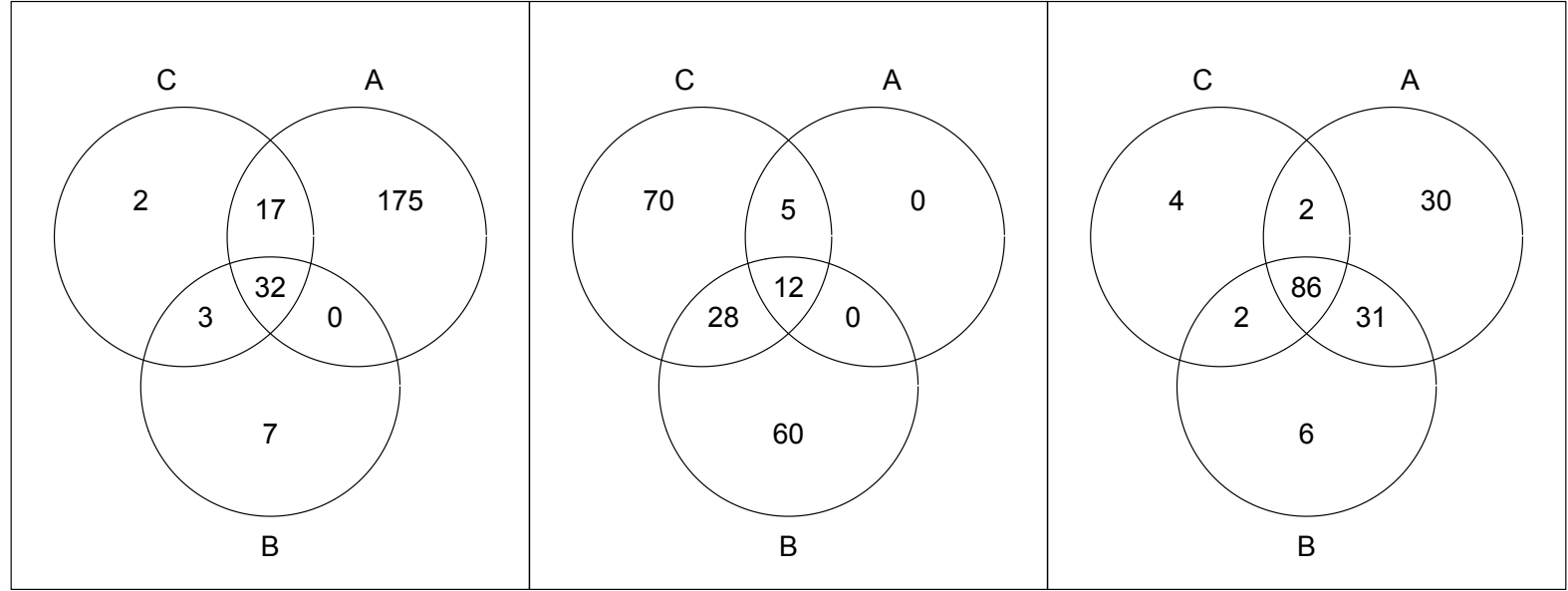

Supplement: Additional file 3 — Number of differentially expressed genes identified in replicate analyses. Numbers of genes reported to be differentially expressed after standard analysis (quantile normalisation) (left), after a standard analysis with mean-centring (middle), and after a standard analysis augmented with the ComBat batch correction (right). A and B refer to the results from independent analyses of the duplicate sample groups while C refers to the results from the pooled duplicate samples. The rows of Venn diagrams illustrate the results with (i) limma, (ii) SAM, (iii) limma using UHRR, and (iv) SAM using UHRR. [file 1471-2164-11-134-S3.PDF]
