# Supplementary material for: Correcting for intra-experiment variation in Illumina BeadChip data is necessary to generate robust gene-expression profiles
Source: BMC Genomics. 2010 Feb 24;11:134. doi: 10.1186/1471-2164-11-134 (PMC2843619; doi:10.1186/1471-2164-11-134)

quantile (p=0.01, FC=1.5)

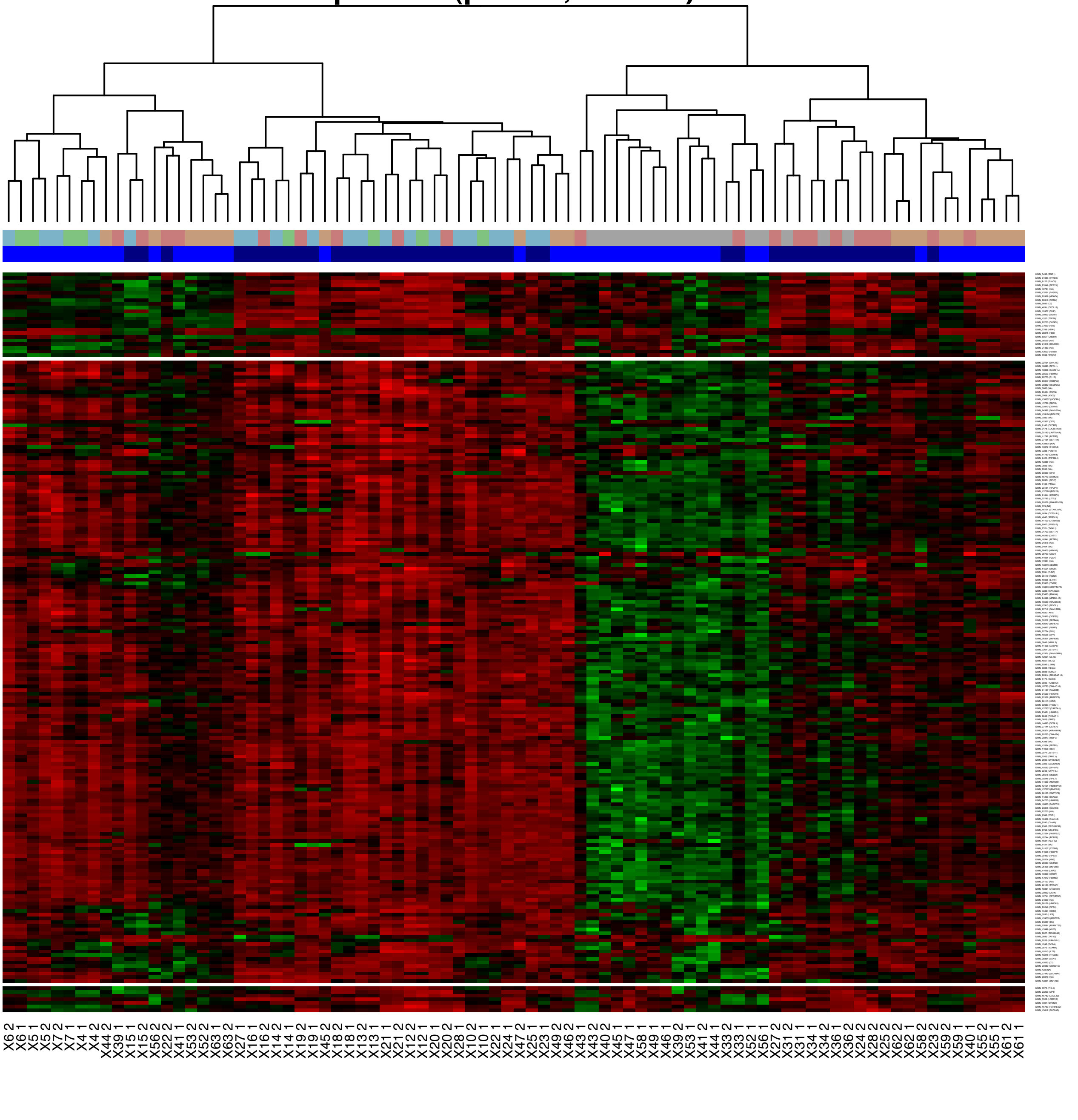

quantile+MeanCenter (p=0.01, FC=1.5)

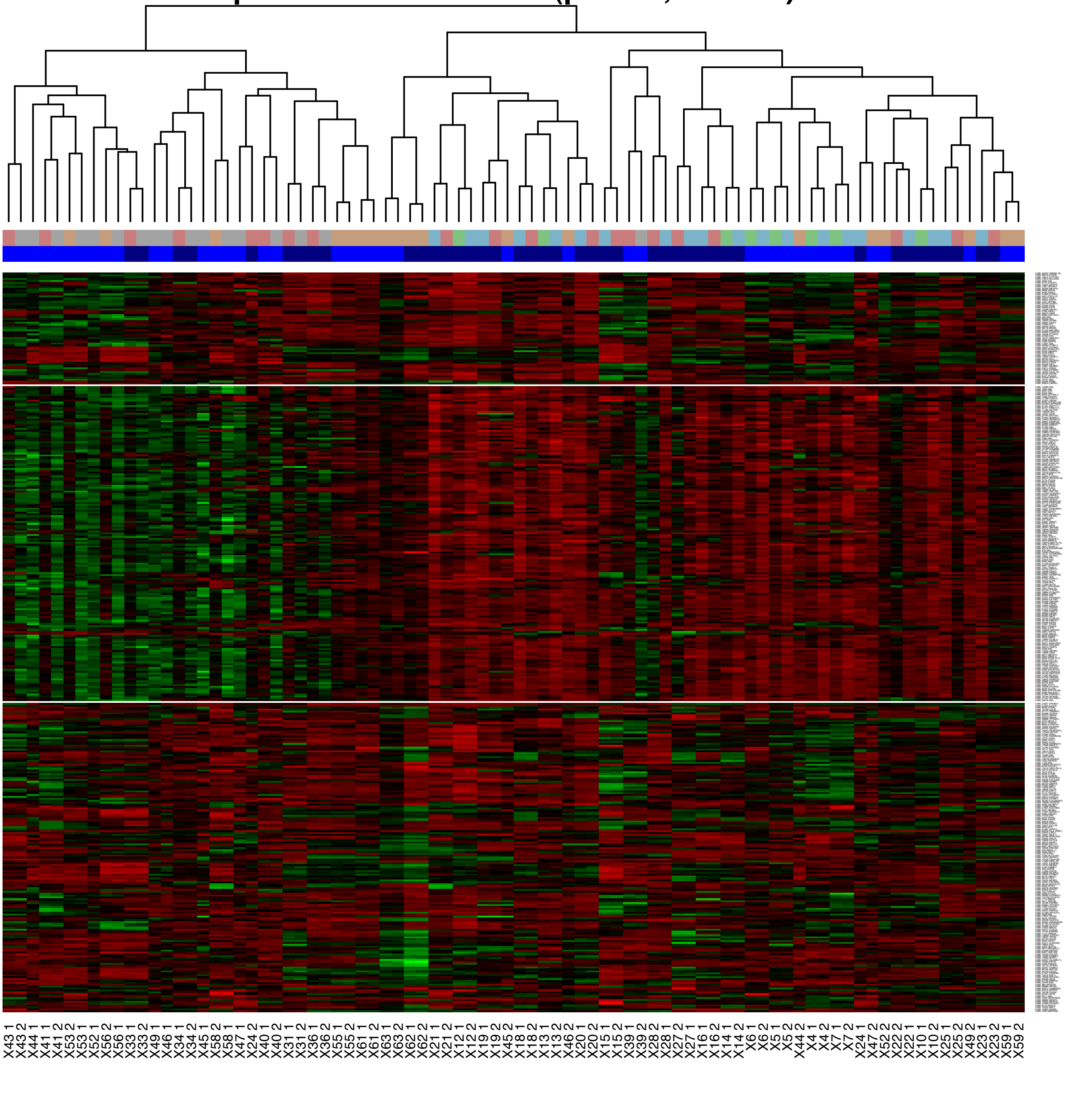

quantile+ComBat (p=0.01, FC=1.5)

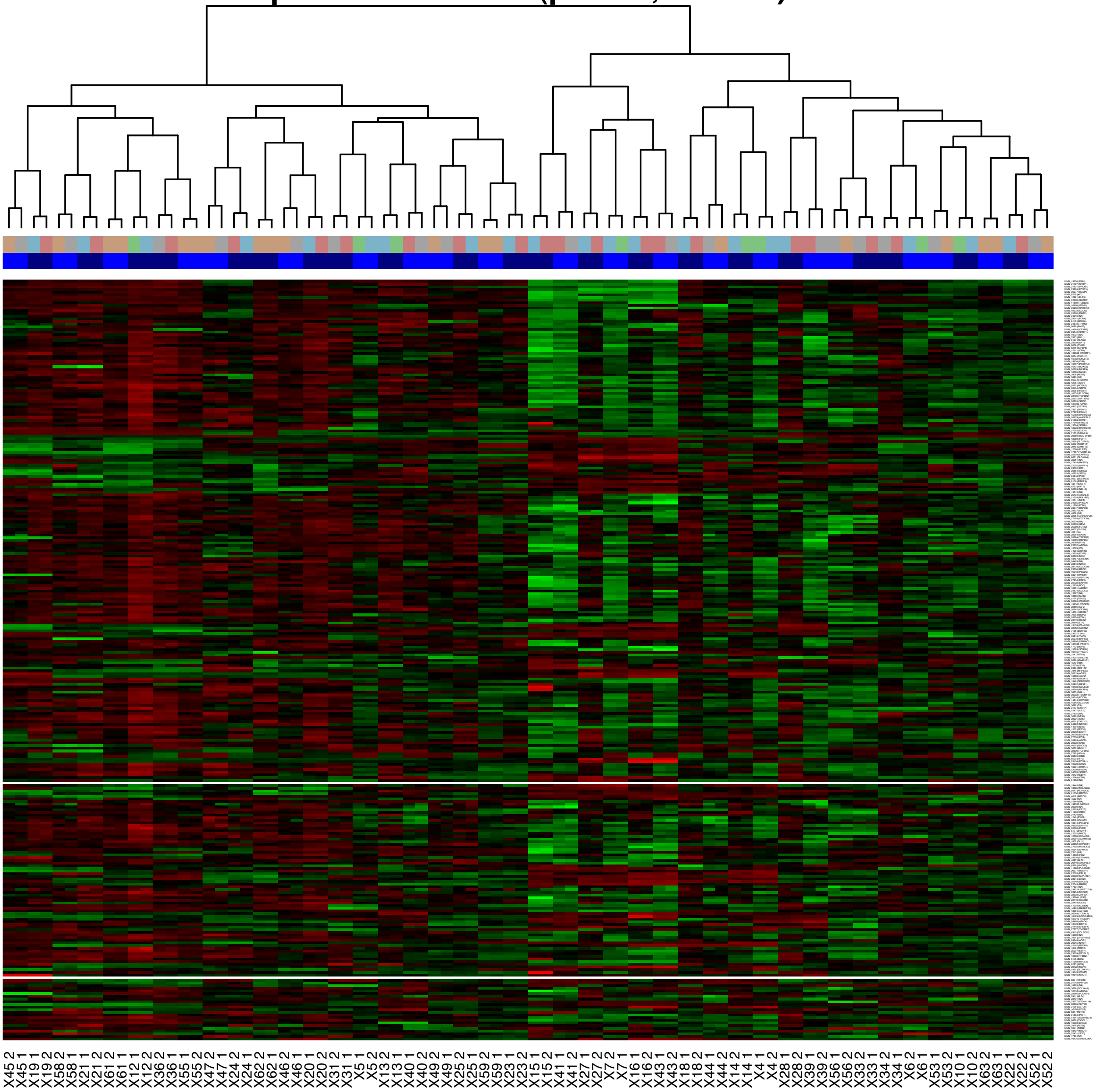

Supplement: Additional file 4 — Heatmaps. Full heatmaps of quantile normalised, quantile normalised plus mean-centred and quantile normalised plus ComBat data, including probe and sample annotations. [file 1471-2164-11-134-S4.PDF]
